# Multi-Level Analyses of Genome-Wide Association Study to Reveal Significant Risk Genes and Pathways in Neuromyelitis Optica Spectrum Disorder

**DOI:** 10.3389/fgene.2021.690537

**Published:** 2021-07-21

**Authors:** Ting Li, He Li, Yue Li, Shu-An Dong, Ming Yi, Qiu-Xia Zhang, Bin Feng, Li Yang, Fu-Dong Shi, Chun-Sheng Yang

**Affiliations:** ^1^Department of Neurology, Tianjin Neurological Institute, Tianjin Medical University General Hospital, Tianjin, China; ^2^Department of Neurology, Tianjin Huanhu Hospital, Tianjin, China; ^3^Department of Anesthesiology, Tianjin Hospital of Integrated Traditional Chinese and Western Medicine, Tianjin, China; ^4^China National Clinical Research Center for Neurological Diseases, Beijing Tiantan Hospital, Capital Medical University, Beijing, China; ^5^Department of Neurology, Barrow Neurological Institute, St. Joseph’s Hospital and Medical Center, Phoenix, AZ, United States

**Keywords:** Neuromyelitis optica spectrum disorder, gene sets, gene differential expression, Genome-wide association study, pathway

## Abstract

**Background:**

Neuromyelitis optica spectrum disorder (NMOSD) is an inflammatory disease of the central nervous system and it is understandable that environmental and genetic factors underlie the etiology of NMOSD. However, the susceptibility genes and associated pathways of NMOSD patients who are AQP4-Ab positive and negative have not been elucidated.

**Methods:**

Secondary analysis from a NMOSD Genome-wide association study (GWAS) dataset originally published in 2018 (215 NMOSD cases and 1244 controls) was conducted to identify potential susceptibility genes and associated pathways in AQP4-positive and negative NMOSD patients, respectively (132 AQP4-positive and 83 AQP4-negative).

**Results:**

In AQP4-positive NMOSD cases, five shared risk genes were obtained at chromosome 6 in AQP4-positive NMOSD cases by using more stringent *p*-Values in both methods (*p* < 0.05/16,532), comprising CFB, EHMT2, HLA-DQA1, MSH5, and SLC44A4. Fifty potential susceptibility gene sets were determined and 12 significant KEGG pathways were identified. Sixty-seven biological process pathways, 32 cellular-component pathways, and 29 molecular-function pathways with a *p*-Value of <0.05 were obtained from the GO annotations of the 128 pathways identified. In the AQP4 negative NMOSD group, no significant genes were obtained by using more stringent *p*-Values in both methods (*p* < 0.05/16,485). The 22 potential susceptibility gene sets were determined. There were no shared potential susceptibility genes between the AQP4-positive and negative groups, furthermore, four significant KEGG pathways were also identified. Of the GO annotations of the 165 pathways identified, 99 biological process pathways, 37 cellular-component pathways, and 29 molecular-function pathways with a *p*-Value of <0.05 were obtained.

**Conclusion:**

The potential molecular mechanism underlying NMOSD may be related to proteins encoded by these novel genes in complements, antigen presentation, and immune regulation. The new results may represent an improved comprehension of the genetic and molecular mechanisms underlying NMOSD.

## Introduction

Neuromyelitis optica spectrum disorder (NMOSD) is an inflammatory disease of the central nervous system that predominantly affects the spinal cord and optic nerves (Wingerchuk et al., 2015). The estimated prevalence is 0.5 to 10 persons (predominantly women) per population of 100,000 (Hor et al., 2020), especially high incidence in Asia, and with an incidence of 0.445/100,000 (0.433-0.457) in China (Tian et al., 2020). The pathogenic role of anti-aquaporin 4 autoantibody (AQP4-Ab) has been established in previous studies. Complement-dependent cytotoxicity and antibody-dependent cell-mediated cytotoxicity have been reported (Wingerchuk et al., 2007). Progression in NMOSD research has led to the deduction that environmental and genetic factors underlie the etiology of NMOSD, environmental factors including changes in the diet, exposure to infections, stressful life events, seasonal variation and so on (Akaishi et al., 2020; Hor et al., 2020; Rafiee et al., 2020).

Recent studies on genome-wide association (GWAS) of NMOSD have amplified the understanding of NMOSD. Previous studies revealed that a common promoter single-nucleotide polymorphisms (SNP) in CYP7A1 has a protective/gene dose-dependent effect on the risk of NMO (Kim et al., 2010). Recently, Estrada et al. (2018) identified two independent SNP (rs1150757 and rs28383224) in the major histocompatibility complex (MHC) region associated with AQP4-Ab positive NMOSD, however, the susceptibility genes and associated pathways of NMOSD patients with AQP4-Ab positive and negative have not been elucidated.

**FIGURE 1:**
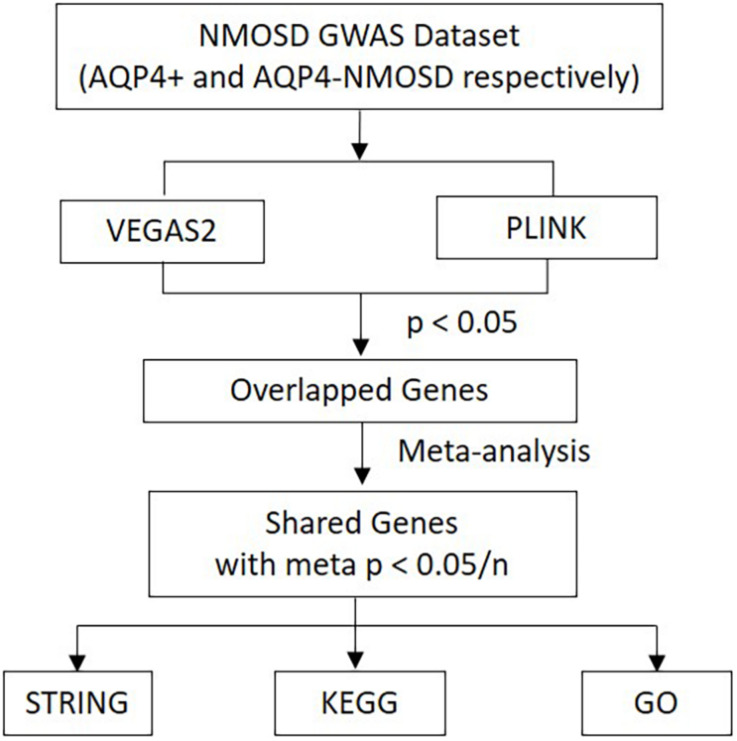
Flow diagram of the three-phase analysis design. In phase I, two new gene-based tests for the GWAS approach, called VEGAS2 and PLINK, were used to conduct a gene-based association study. In phase II, Gene sets identified with meta *p* < 0.05/n were carried forward to the next phase. In phase III, protein-protein association networks were performed by STRING. Meanwhile, KEGG pathways and GO analysis were carried out for these NMOSD susceptibility genes. VEGAS2 and PLINK are two kinds of new gene-based tests. “n” represents the number of genes shared by the VEGAS2 and PLINK tests.

As a useful complementary approach for SNP-GWAS, gene-based tests play an increasing role in genetics research. Studies have shown that gene-based tests can be more powerful than SNP-GWAS approach and suitable for integrating the results of pathway tests from GWAS (Liu et al., 2010). Secondary analysis from a NMOSD-GWAS dataset originally published in 2018 (215 NMOSD cases and 1244 controls) was conducted to identify potential susceptibility genes and associated pathways of NMOSD (Estrada et al., 2018). The original data was obtained at the NHGRI-EBI GWAS Catalog^1^. In phase I, more stringent *p*-Values were used to determine the intersection of two new gene-based tests for the GWAS approach, called Versatile Gene-based Association Study-2 version 2 (VEGAS2) and PLINK, which were used to conduct a gene-based association study in AQP4-positive (*p* < 0.05/16,532) and negative (*p* < 0.05/16,485) NMOSD patients, respectively. Additionally, for exploring more potential genes, we used *p* < 0.05 to get the share genes of these two methods. In phase II, gene sets identified with meta *p* < 0.05/n (“n” represents the number of genes shared by the VEGAS2 and PLINK tests) were carried forward to the next phase. In phase III, protein-protein association networks were performed by STRING, meanwhile, KEGG pathways and GO analysis were conducted for the NMOSD susceptibility genes to gain further insights into the genes (Figure 1).

In AQP4-positive NMOSD cases, where more stringent *p*-Values were used in both methods, five shared risk genes were obtained at chromosome 6 (*p* < 0.05/16,532), comprising CFB, EHMT2, HLA-DQA1, MSH5, and SLC44A4. Furthermore, we determined 50 potential susceptibility gene sets of AQP4-positive NMOSD, and the top 5 of these genes were the same as the genes obtained using more stringent *p*-Values after which 12 significant KEGG pathways (*p* < 0.05) were identified. In the GO annotations of the 128 pathways identified, 67 biological process pathways, 32 cellular-component pathways, and 29 molecular-function pathways with a *p*-Value of <0.05 were obtained. No significant genes were obtained by using more stringent *p*-Values in both methods in the AQP4-negative NMOSD group (*p* < 0.05/16,485). The 22 potential susceptibility gene sets were determined. There were no shared potential susceptibility genes between the AQP4-positive and negative groups, furthermore, four significant KEGG pathways were also identified. Of the GO annotations of 165 pathways identified, 99 biological process pathways, 37 cellular-component pathways, and 29 molecular-function pathways with a *p*-Value of <0.05 were obtained. In short, the new results may represent significant steps toward defining the genetic mechanism underlying the association of NMOSD.

## Materials and Methods

### Samples

The original data were obtained from the NHGRI-EBI GWAS Catalog see text footnote 1. The large-scale NMOSD-GWAS dataset that comprises 215 NMOSD cases (132 AQP4-positive and 83 AQP4-negative) and 1244 controls of European ancestry was analyzed. The originally published data in 2018 used 2006 NMO diagnostic criteria that require optic neuritis and transverse myelitis plus two of the following three supportive elements: (1) longitudinally extensive lesions (≥3 vertebral segments in length); (2) magnetic resonance imaging of the brain with normal findings or with findings inconsistent with MS; and (3) NMO-IgG seropositivity. After subjecting the dataset to certain quality-control methods, 7,138,498 autosomal SNPs were available for genetic analysis. Please refer to the original text for more details (Estrada et al., 2018). To investigate whether there are genetic differences between AQP4-positive and AQP4 negative NMOSD, gene-based association study in AQP4-positive and negative NMOSD patients was conducted.

### Data Analysis

1. Gene-based test
  The first gene-based test method used was a versatile gene-based association study (VEGAS2^2^) developed by Aniket Mishra and Stuart Macgregor (Mishra and Macgregor, 2015). VEGAS2 distributes SNPs to every gene of 17,787 autosomes in terms of the location on the UCSC Genome Browser hg18 group. The method integrates the overall information of the gene and its SNPs while also illustrating their details such as the sizes of genes and the linkage disequilibrium (LD) between SNPs. ± 50 kb of 5′ and 3′. UTRs, which is defined as the inclusion of SNPs screening within genes. Subsequently, the association *p*-Values of SNPs within a given gene are converted to upper-tail chi-squared statistics with one degree of freedom (df) (Liu et al., 2010). The statistic of this gene-based test is the total of all χ^2^ 1 df statistics in the given gene. This method uses the multivariate normal simulations to illustrate the LD structure of SNPs within the given gene with reference to the HapMap2 genotype information (Liu et al., 2010). Simulations of 10^3^ are first processed. If the out-coming empirical *p*-Value is less than 0.1 then 10^4^ simulations will be processed. If the empirical *p*-Value of 10^4^ simulations is less than 0.001, the process will perform 10^6^ simulations. Due to the calculated reasons, no more simulations will be processed if the empirical *p*-Value is 0. More stringent *p*-Values have been used for both methods in AQP4-positive NMOSD cases (*p* < 0.05/16,532) and in the AQP4-negative NMOSD group (*p* < 0.05/16,485). For exploring more potential genes, *p* < 0.05 was used to get the intersection of two methods and an empirical *p*-Value of 0 from 10^6^ simulations can be explained as *p* < 10^–6^, which surpasses *p*-Value < 2.8 10^–6^ (≈0.05/17,787(the autosomal genes’ amount)) (Liu et al., 2010). Gene-based, test method based, user-friendly bioinformatics tools, like PLINK which was a set-based analysis, were used and this method combined a meta-analysis process and PLINK to calculate the data of GWAS (Moskvina et al., 2011). The theoretical description of this method is detailed in the previously published literature (Liu et al., 2017). The PLINK is an open-source C/C++ whole-genome association study software^3^ (Purcell et al., 2007). Based on the HapMap data available in PLINK, “plink –bfile hapmap_CEU_r23a –set-screen nmo.txt –make-set glist.dat” was conducted for gene-wise calculations (Moskvina et al., 2011). In the code above, “nmo.txt” is the file with SNP IDs and their *p*-Value in NMO GWAS data, other files are provided by the software (Moskvina et al., 2011).
2. Meta-analysis
  Fisher’s method was used to integrate the *p*-Values of common risk genes derived from two gene-based tests. Fisher’s method is a simple meta-analysis approach for significance computation (Kippola and Santorico, 2010). The statistics of Fisher’s method are computed as:
  (2-1) $${\text{x}}^{2}=-2\,\sum_{i=1}^{k}\ln\left(p_{i}\right)$$
  In 2-1, *i* is the number of studies, *p*_*i*_ is the *p*-Values of the gene and *k* is the sum count of studies. This formula follows the chi-squared distribution with 2 *k*df.
3. Protein-protein association networks performed by string
  STRING (version 11) was performed for functional partnerships and interactions between the proteins encoded by the overlapped NMOSD genes by two gene-based tests^4^. The STRING database plans on assembling and integrating the proteins association information (Szklarczyk et al., 2017). The subtypes of associations in STRING are direct (physical) and indirect (functional) interactions. The source of the STRING database includes assembling and evaluating obtainable tested data from known protein complexes and pathways, co-expression study, inter-genomes shared selective signals, scientific article text-mining, and interaction knowledge calculated transfer between organisms (Szklarczyk et al., 2017). The association network of STRING provides much information of proteins including annotations, cross-links, domain structures, 3D protein structures, and their types of interaction (Szklarczyk et al., 2015). The new version (v11) of STRING updates the organisms’ amount to 5,090 and increased the capacity of the input to the genome-wide level (Szklarczyk et al., 2019). In a study of the genome-wide dataset, the subsets can be visualized as association networks and conducted as enrichment analysis. Furthermore, STRING actualized novel classification systems for enrichment analysis in addition to classical systems such as Gene Ontology and KEGG (Szklarczyk et al., 2019).
4. Pathway-based test
  In the study, widely used online assessment analysis tools were highlighted for enrichment analysis, WebGestalt 2019^5^ (Liao et al., 2019). A hypergeometric test was conducted to explore an over-representation of the screened genes among the genes in a given pathway (Wang et al., 2013). In this pathway, the *p*-Values of more than J disease-related genes were observed and computed by the following formula:
  (4-1) $$P=1-\sum_{\text{i}=0}^{J}\frac{\left(\begin{array}{c} \text{S}\\ \text{i} \end{array}\right)\,\left(\begin{array}{c} \text{N–S}\\ \text{m–i} \end{array}\right)}{\left(\begin{array}{c} \text{N}\\ \text{m} \end{array}\right)}$$
  In 4-1, m is the total number of target genes related to a given disease, N is the number of reference genes, S is the number of genes in the designated pathways. The pathway selection interval was set between 20 and 300 genes to avoid bias caused by testing an extremely narrow or broad enrichment of genes on the pathway (Liao et al., 2019). According to the result, the pathways with *p*-Value < 0.05 were regarded as risk pathways.

## Results

### Gene-Based Test of NMOSD GWAS Dataset

In the AQP4-positive NMOSD cases, 6,804,718 NMOSD SNPs were mapped to 21,275 and 16532 genes, respectively, using VEGAS2 and PLINK. More stringent *p*-Values were used in both methods, five shared risk genes were obtained at chromosome 6 (*p* < 0.05/16,532), comprising CFB, EHMT2, HLA-DQA1, MSH5, and SLC44A4 (Table 1 and detailed results are listed in Supplementary Table 1). For exploring more potential genes, *p* < 0.05 was used to get the intersection of two methods after which 961 genes were identified using VEGAS2, and 544 genes using PLINK with a significance of *p* < 0.05, 315 common genes were revealed by VEGAS2 and PLINK (Supplementary Table 2), of which 50 gene sets passed the Bonferroni-corrected statistical test at a significance of meta *p* < 1.59 × 10^–4^ (*p* = 0.05/315) (Supplementary Table 2). The 50 potential susceptibility gene sets of AQP4-positive NMOSD are shown in Table 2 and the top 5 of these genes were same with the ones obtained with more stringent *p*-Values (*p* < 0.05/16,532).

**TABLE 1:** Susceptibility genes by using more stringent *p*-Values and the intersection of VEGAS2 and PLINK in AQP4-positive NMOSD.

Gene set	Position	nSNPs	*P*-value
**VEGAS2**			
ATP6V1G2	chr6:31620699..31621843	11	1E-06
ATP6V1G2-DDX39B	chr6:31530219..31546848	20	1E-06
CFB*	chr6:32022003..32027809	18	1E-06
CLIC1	chr6:31810689..31812273	4	1E-06
EHMT2*	chr6:31956199..31972526	16	1E-06
HLA-DQA1*	chr6:32717784..32717784	22	1E-06
MB21D1	chr6:73423711..73452297	29	1E-06
MSH5*	chr6:31816126..31837338	34	1E-06
MSH5-SAPCD1	chr6:31739948..31764850	36	1E-06
SLC44A4*	chr6:31942176..31954213	44	1E-06
STK19_1	chr6:31971175..31981446	10	1E-06
TNXB_2	chr6:32041153..32109338	85	1E-06
SLC17A5	chr6:74361136..74419621	334	2E-06
**PLINK**			
AGER	chr6:32257794..32259972	5	1.09E-06
BTNL2	chr6:32470908..32482618	35	1.43E-06
C6orf10	chr6:32368537..32447625	213	1.88E-09
C6orf136	chr6:30726885..30726885	1	7.09E-08
C6orf27	chr6:31841629..31850569	4	6.22E-07
C6orf48	chr6:31912625..31915519	4	1E-08
CFB*	chr6:32022003..32027809	15	1.2E-06
EGFL8	chr6:32242488..32242488	1	1.9E-08
EHMT2*	chr6:31956199..31972526	10	1.81E-09
HLA-DQA1*	chr6:32717784..32717784	1	3.29E-09
HLA-DRA	chr6:32515687..32520787	37	8.77E-10
MSH5*	chr6:31816126..31837338	19	3.78E-10
PBX2	chr6:32262263..32265617	4	3.54E-07
PRRT1	chr6:32225949..32225949	1	2.21E-08
RNF5	chr6:32254470..32256381	6	9.36E-08
SKIV2L	chr6:32035321..32045016	17	6.39E-07
SLC44A4*	chr6:31942176..31954213	19	2.82E-06
STK19	chr6:32048876..32055439	7	2.24E-13

NSNP: number of single nucleotide polymorphisms. * represents the intersection of VEGAS2 and PLINK.

**TABLE 2:** Comparison of significant shared gene sets in AQP4-positive NMOSD produced with VEGAS2 and PLINK.

Gene set	Position	PLINK		VEGAS2		
		NSNP	*P*-value	NSNP	*P*-value	P meta
MSH5	chr6:31816126..31837338	19	3.78E-10	34	1.00E-06	1.38E-14
EHMT2	chr6:31956199..31972526	10	1.81E-09	16	1.00E-06	6.31E-14
HLA-DQA1#	chr6:32717784..32717784	1	3.29E-09	22	1.00E-06	1.13E-13
CFB	chr6:32022003..32027809	15	1.20E-06	18	1.00E-06	3.42E-11
SLC44A4	chr6:31942176..31954213	19	2.82E-06	44	1.00E-06	7.77E-11
C6orf10	chr6:32368537..32447625	213	1.88E-09	14	0.0027	1.37E-10
CLIC1	chr6:31810689..31812273	2	7.76E-06	4	1.00E-06	2.06E-10
SLC17A5	chr6:74361136..74419621	92	4.78E-06	334	2.00E-06	2.52E-10
HIST1H1B	chr6:27943197..27943251	2	8.83E-06	12	1.20E-05	2.54E-09
HIST1H2BL	chr6:27883653..27883676	2	2.57E-05	6	2.10E-05	1.21E-08
HIST1H2AL	chr6:27941153..27941153	1	2.66E-05	9	2.30E-05	1.36E-08
ATP6V1G2	chr6:31620699..31621843	5	0.003494	11	1.00E-06	7.15E-08
ZSCAN16	chr6:28200582..28205172	8	7.64E-05	14	5.40E-05	8.38E-08
BTN3A2	chr6:26473574..26486128	39	0.000377	89	2.20E-05	1.62E-07
EEF1A1	chr6:74283039..74286513	7	9.38E-05	20	9.40E-05	1.72E-07
MTO1	chr6:74228188..74266790	17	0.000618	78	2.30E-05	2.71E-07
VARS	chr6:31856799..31870822	5	1.10E-05	2	0.00213	4.36E-07
C6orf15	chr6:31187055..31188078	9	0.000135	24	0.000181	4.54E-07
ARSI	chr5:149656490..149660452	3	0.000152	18	0.000197	5.48E-07
ZFP57	chr6:29750488..29756485	16	0.000276	17	0.000148	7.37E-07
BTN2A1	chr6:26566244..26576639	20	0.003007	66	3.60E-05	1.84E-06
C2	chr6:32003952..32019988	12	0.001923	41	8.30E-05	2.66E-06
LTA	chr6:31648120..31648763	5	0.001067	8	0.000202	3.52E-06
HIST1H3J	chr6:27966400..27966400	1	0.000498	6	0.000467	3.79E-06
PSORS1C1	chr6:31190939..31215712	65	0.005206	149	6.40E-05	5.3E-06
GLS	chr2:191457280..191537657	43	0.000938	108	0.000379	5.63E-06
CDSN	chr6:31190939..31192771	17	0.001358	35	0.000402	8.42E-06
APPL1	chr3:57238434..57282001	17	0.000839	63	0.000788	1.01E-05
NFKBIL1	chr6:31623742..31633891	12	0.003355	32	0.000241	1.22E-05
ZNF165	chr6:28154652..28163474	8	0.003189	29	0.00042	1.95E-05
ZKSCAN3	chr6:28426460..28441375	24	0.008439	64	0.000175	2.13E-05
HSPA1A	chr6:31891486..31891486	1	0.000957	2	0.00157	2.16E-05
C6orf203	chr6:107458491..107478905	17	0.000463	87	0.00427	2.79E-05
ZMYND19	chr9:139597070..139602897	4	0.000315	19	0.00638	2.83E-05
EIF3F	chr11:7965996..7973800	16	0.004203	35	0.000498	2.95E-05
PSORS1C2	chr6:31213289..31215066	12	0.004212	16	0.000612	3.57E-05
HRASLS2	chr11:63078313..63086972	5	0.003647	14	0.000846	4.22E-05
KLHL30	chr2:238715095..238725505	11	0.000507	54	0.0069	4.75E-05
CAPN2	chr1:221967442..222030091	65	0.002096	206	0.00187	5.27E-05
CDC16	chr13:114021570..114055715	19	0.003988	90	0.00103	5.51E-05
PKN3	chr9:130508561..130522288	2	0.001899	13	0.00222	5.64E-05
TBCB	chr19:41301630..41306302	5	0.000416	25	0.015898	8.55E-05
TJP2	chr9:70980117..71059730	108	0.007071	418	0.001	9.09E-05
TAP2	chr6:32897785..32914439	65	0.006917	112	0.00111	9.81E-05
RBBP6	chr16:24458431..24490907	30	0.005909	75	0.00134	0.000101
CYP2A6	chr19:46042434..46046373	3	0.001634	26	0.00522	0.000108
PSMB9	chr6:32930164..32934963	14	0.002805	23	0.00336	0.000118
TCP10	chr6:167715748..167716814	4	0.004822	28	0.00241	0.000144
POLA2	chr11:64786765..64821494	15	0.003271	36	0.00391	0.000157
LST1	chr6:31662586..31664560	6	0.0259	7	0.000496	0.000158

*NSNP: number of single nucleotide polymorphisms. # represents identified susceptibility genes.*

In the AQP4-negative NMOSD group, 6,423,746 NMOSD SNPs were mapped to 21,170 and 16485 genes, respectively using VEGAS2 and PLINK. More stringent *p*-Values were used in both methods, no significant genes were determined (*p* < 0.05/16,485). To explore more potential genes, *p* < 0.05 was used to get the intersection of two methods after which 988 genes were identified using VEGAS2 and 473 genes using PLINK with a significance of *p* < 0.05, 305 common genes were identified (Supplementary Table 2) of which 22 gene sets passed the Bonferroni-corrected statistical test at a significance of meta *p* < 1.63 × 10^–4^ (*p* = 0.05/305). The 22 potential susceptibility gene sets of AQP4 negative NMOSD are shown in Table 3. Shared susceptibility genes between the AQP4-positive and negative groups were absent.

**TABLE 3:** Comparison of significant shared gene sets in AQP4 negative NMOSD produced with VEGAS2 and PLINK.

Gene set	Position	VEGAS2		PLINK		
		NSNP	*P*-value	NSNP	*P*-value	P meta
RGS1	chr1:190811722..190815166	9	0.001563	25	0.000215	5.35E-06
COL8A2	chr1:36338171..36338204	2	4.32E-05	15	0.015598	1.02E-05
AGTPBP1	chr9:87354033..87545005	115	0.000611	413	0.00196	1.75E-05
SYT9	chr11:7232814..7446395	376	0.02569	743	0.000047	1.77E-05
SGIP1	chr1:66772691..66982406	374	0.002918	654	0.000461	1.95E-05
CEACAM4	chr19:46817929..46824888	10	0.03613	31	0.000043	2.23E-05
INTS5	chr11:62177128..62177128	1	0.000506	5	0.00558	3.89E-05
RAD51AP2	chr2:17557392..17562159	7	0.003099	36	0.000931	3.97E-05
CDR2L	chr17:70496373..70513013	6	0.000389	20	0.00889	4.7E-05
ZNF652	chr17:44728416..44791811	39	0.005543	148	0.000784	5.8E-05
WDR86	chr7:150709151..150736999	23	0.008985	101	0.000599	7.07E-05
HSD11B1	chr1:207927291..207973298	28	0.005835	76	0.000976	7.45E-05
SYCE1	chr10:135217645..135231917	54	0.001929	100	0.00354	8.81E-05
SSFA2	chr2:182469362..182502779	16	0.003418	48	0.00205	9.02E-05
WT1	chr11:32366048..32412429	65	0.002659	159	0.00276	9.41E-05
RAX2	chr19:3720253..3720899	4	0.001959	16	0.004	1E-04
CAPG	chr2:85475570..85489706	11	0.009872	77	0.000851	0.000107
PIGX	chr3:197926529..197946898	12	0.003838	75	0.00228	0.000111
RPS3	chr11:74788237..74794135	4	0.001819	31	0.00564	0.000128
PGRMC2	chr4:129414746..129427709	5	0.003674	19	0.0028	0.000128
TRAF3IP3	chr1:207996202..208020329	32	0.00363	61	0.0033	0.000148
PTPLAD1	chr15:63611672..63657183	24	0.005597	89	0.00231	0.000158

*NSNP: number of single nucleotide polymorphisms.*

#### Protein-Protein Association Network Analysis of NMOSD Susceptibility Genes

**FIGURE 2:**
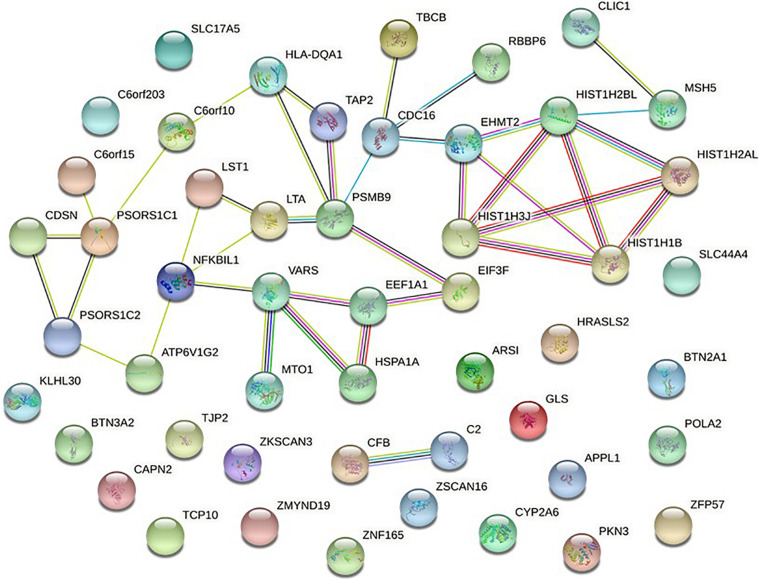
Network of known and predicted interactions between proteins encoded by NMOSD potential susceptibility genes were identified by GWAS of AQP4-positive NMOSD. Network nodes represent the proteins produced by a single, protein-coding gene locus. Edges represent protein-protein associations meant to be specific and meaningful. In STRING, blue edge represents protein-protein associations with known interactions from curated databases, purple edge represents protein-protein associations with known interactions experimentally determined, green edge represents protein-protein associations with predicted interactions with gene neighborhood, red edge represents protein-protein associations with predicted interactions with gene fusions, Navy blue edge represents protein-protein associations with predicted interactions with gene co-occurrence, and other associations with text mining (yellow edge), co-expression (black edge), and protein homology (lavender edge).

**FIGURE 3:**
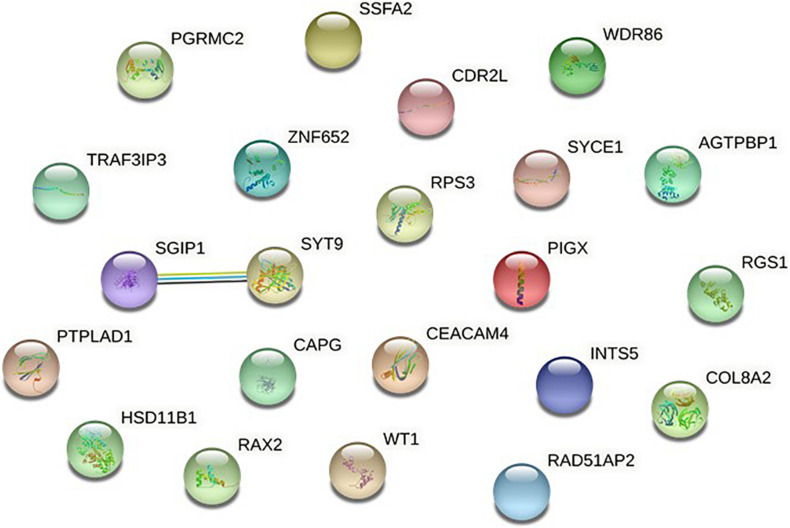
Network of known and predicted interactions between proteins encoded by NMOSD potential susceptibility genes were identified by GWAS of AQP4-negative NMOSD.

To further verify these findings, a protein-protein interaction network analysis was conducted for AQP4-positive and negative NMOSD potential susceptibility genes. Interestingly, significant connectivity among proteins encoded by these NMOSD susceptibility genes was highlighted according to the protein-protein interaction network in STRING (version 11.0) (Figures 2, 3).

#### Pathway Analysis of NMOSD-GWAS Dataset

**FIGURE 4:**
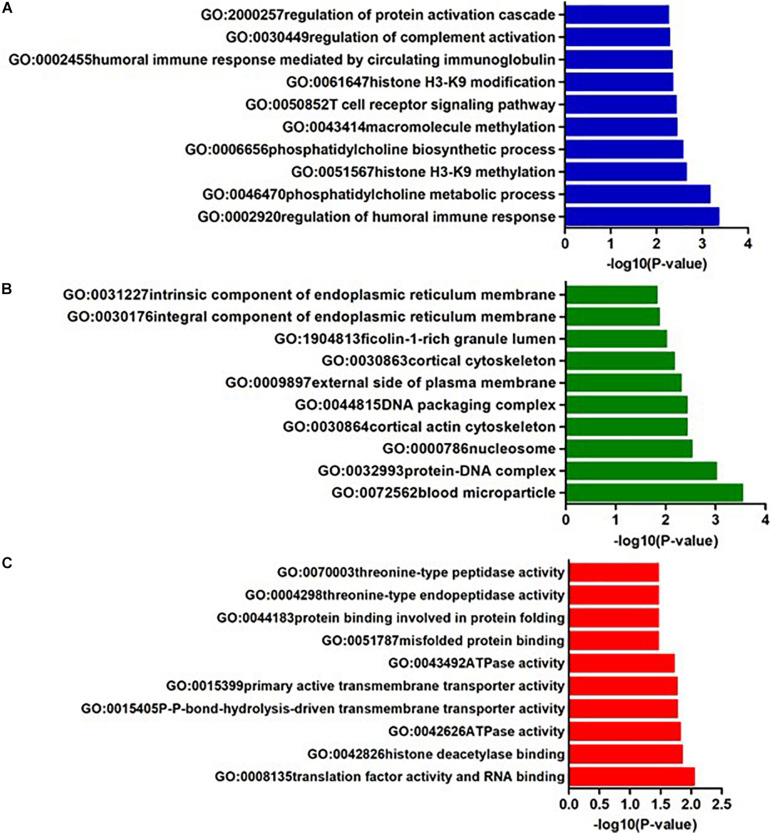
The top ten GO annotations of AQP4-positive NMOSD, **(A)** is for biological process, **(B)** is for molecular function, and **(C)** is for cellular component.

In AQP4-positive NMOSD, KEGG pathway analysis was conducted on the 50 potential susceptibility genes, and 12 significant KEGG pathways were identified. (*p* < 0.05). The 12 KEGG pathways fall into seven categories: (1) Immune disease (*n* = 2) : systemic lupus erythematosus (SLE) (hsa05322) and Rheumatoid arthritis (RA) (hsa05323), (2) Immune system (*n* = 2): antigen processing and presentation (hsa04612), complement and coagulation cascades (hsa04610), (3) Infectious disease (*n* = 4): *Staphylococcus aureus* infection (hsa05150), *Vibrio cholerae* infection (hsa05110), Leishmaniasis (hsa05140) and herpes simplex infection (hsa05168), (4) Endocrine and metabolic disease (*n* = 1): Type I diabetes mellitus (hsa04940), (5) Transport and catabolism (*n* = 1): Phagosome (hsa04145), (6) Substance dependence (*n* = 1): Alcoholism (hsa05034), (7) Longevity regulating pathway (*n* = 1): Longevity regulating pathway (hsa04211) (Table 4). In KEGG, genes associated with immune disease and system include HLA-DQA1, HIST1H2BL, HIST1H2AL, C2, CFB, HSPA1A, TAP2, HIST1H3Jand ATP6V1G2. In GO annotations of the 128 pathways identified, 67 biological process pathways, 32 cellular-component pathways, and 29 molecular-function pathways with a *p*-Value of <0.05 were obtained. The top 10 of GO annotations are illustrated in Figure 4, and detailed results are listed in Supplementary Table 2. Regarding the GO analysis, regulation of humoral immune response (GO:0002920), protein processing (GO:0016485), regulation of complement activation (GO:0030449), regulation of protein activation cascade (GO:2000257) and complement activation (GO:0006956) were associated with C2 and CFB locus. Histone H3K9 methylation (GO:0051567) and histone methyltransferase activity (H3K27 and H3K9) (GO:0046976 and GO:0046976) were associated with EHMT2 and HIST1H1B locus; phosphatidylcholine metabolic process (GO:0046470), phosphatidylcholine biosynthetic process (GO:0006656) and vitamin transmembrane transporter activity (GO:0090482) were associated with SLC44A4 locus; DNA repair complex (GO:1990391) was associated with MSH5 locus; pathways related to antigen processing, presentation (GO:0002478, GO:0019884, and GO0048002) and MHC protein complex (GO:0042611) are also associated with HLA-DQA1 locus.

**TABLE 4:** Significant KEGG pathways of AQP4-positive and negative with *P* < 0.05 by pathway analysis of NMOSD GWAS.

Pathway ID	Pathway name	Pathway classification	*P*-value	Related genes
**AQP4-positive**				
hsa05322	Systemic lupus erythematosus	Immune disease	6.62E-05	HLA-DQA1; HIST1H2BL; HIST1H2AL; C2; HIST1H3J
hsa05150	Staphylococcus aureus infection	Infectious disease	8.17E-04	HLA-DQA1; CFB; C2
hsa04612	Antigen processing and presentation	Immune system	0.002058	HLA-DQA1; HSPA1A; TAP2
hsa04940	Type I diabetes mellitus	Endocrine and metabolic disease	0.008931	HLA-DQA1; LTA
hsa05110	Vibrio cholerae infection	Infectious disease	0.011943	ATP6V1G2; TJP2
hsa04145	Phagosome	Transport and catabolism	0.013689	HLA-DQA1; ATP6V1G2; TAP2
hsa05134	Legionellosis	Infectious disease	0.014332	EEF1A1; HSPA1A
hsa05034	Alcoholism	Substance dependence	0.021444	HIST1H2BL; HIST1H2AL; HIST1H3J
hsa05168	Herpes simplex infection	Infectious disease	0.023038	HLA-DQA1; LTA; TAP2
hsa04610	Complement and coagulation cascades	Immune system	0.028318	CFB; C2
hsa04211	Longevity regulating pathway	Aging	0.03527	EHMT2; APPL1
hsa05323	Rheumatoid arthritis	Immune disease	0.035998	HLA-DQA1; ATP6V1G2
**AQP4-negative**				
hsa00563	Glycosylphosphatidylinositol (GPI)-anchor biosynthesis	Glycan biosynthesis and metabolism	1.33E-02	PIGX
hsa00140	Steroid hormone biosynthesis	Lipid metabolism	3.18E-02	HSD11B1
hsa00980	Metabolism of xenobiotics by cytochrome P450	Xenobiotics biodegradation and metabolism	4.01E-02	HSD11B1
hsa05204	Chemical carcinogenesis	Cancer	4.32E-02	HSD11B1

**FIGURE 5:**
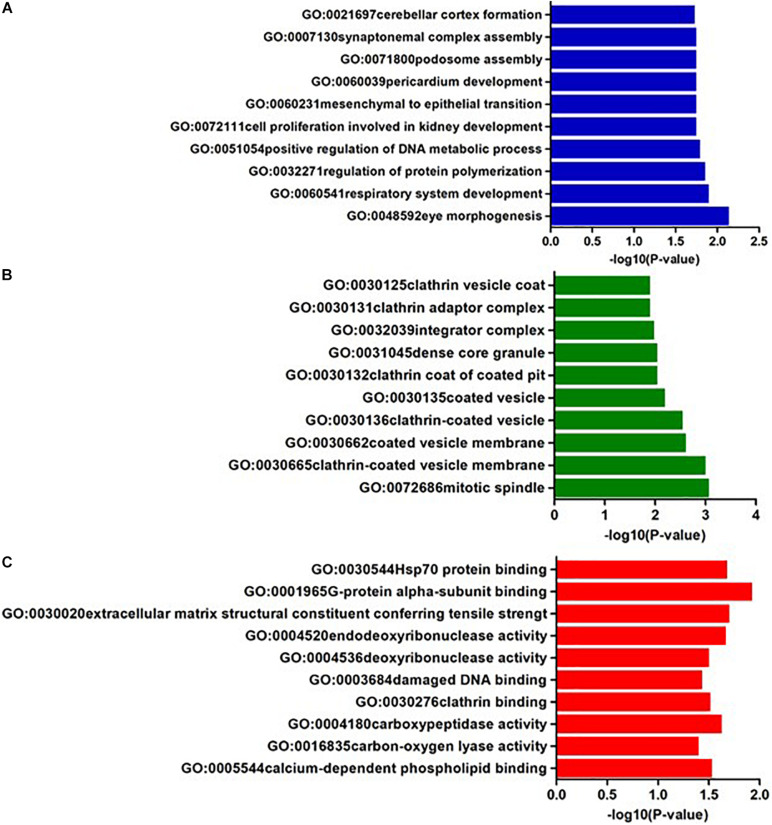
The top ten GO annotations of AQP4- negative NMOSD, **(A)** is for biological process, **(B)** is for molecular function, and **(C)** is for cellular component.

In AQP4 negative NMOSD, seven KEGG pathways were available for the analysis of 22 potential susceptibility genes. Four significant KEGG pathways were highlighted (*p* < 0.05). The 4 KEGG pathways are comprised of four categories: (1) Glycan biosynthesis and metabolism: glycosylphosphatidylinositol (GPI)-anchor biosynthesis (hsa00563), (2) Lipid metabolism: Steroid hormone biosynthesis (hsa00140), (3) Xenobiotics biodegradation and metabolism: metabolism of xenobiotics by cytochrome P450 (hsa00980), (4) Cancer: Chemical carcinogenesis (hsa05204) (Table 4). In GO annotations of the 165 pathways identified, 99 biological process pathways, 37 cellular-component pathways, and 29 molecular-function pathways with a *p*-Value of <0.05 were obtained. The top 10 results of the GO analysis are illustrated in Figure 5, and detailed results are listed in Supplementary Table 4.

## Discussion

Progression in NMOSD research has led to the deduction that environmental and genetic factors underlie the etiology of NMOSD. NMOSD genetic risk is still required to be identified to date. A NMOSD-GWAS dataset comprising 215 NMOSD cases (132 AQP4-positive and 83 AQP4-negative) and 1244 control cases of European ancestry which contain 7,138,498 autosomal SNPs was chosen as regards to the present study. As a useful complementary approach to per SNP-GWAS, gene-based tests play many roles in genetics research. Studies have shown that gene-based to be more powerful than the per SNP-GWAS approach, also, they are suitable for integrating the results of pathway tests from GWAS (Liu et al., 2010).

Recently, Estrada et al. (2018) identified two independent single nucleotide polymorphisms (SNPs) (rs1150757 and rs28383224) in the MHC region associated with AQP4-Ab positive NMOSD. For further verification and as a supplement to Estrada et al’s. (2018) research based on SNP analysis, our study is based on gene analysis and focused on functional comparison. In the study, more stringent *p*-Values were used in both methods and five shared risk genes were obtained at chromosome 6 in AQP4-positive NMOSD cases (*p* < 0.05/16,532), while shared risk genes in the AQP4 negative NMOSD group were absent (*p* < 0.05/16,485). Further, there were 50 and 22 potential susceptibility genes in AQP4-positive and negative NMOSD, respectively. Combining the protein-protein association network analysis and pathway tests, focus was on susceptibility genes that were not only significant in gene-based tests, but also meaningful in the functional analyses. In AQP4-positive NMOSD group, the five risk genes comprised CFB associated with complements, HLA-DQA1 associated with antigen processing, and EHMT2, MSH5, and SLC44A4 associated with immune regulation.

Complement factor B (CFB) localizes to the MHC class III region on chromosome 6 and is a component of the alternative pathway of complement activation. Upon activation of the alternative pathway, CFB is cleaved after which it yields the noncatalytic chain Ba and the catalytic subunit Bb. The active subunit Bb is a serine protease which associates with C3b to form the alternative pathway C3 convertase. Bb is involved in the proliferation of preactivated B lymphocytes, while Ba inhibits their proliferation. Previous studies suggest that CFB may contribute to SLE (Gateva et al., 2009). Similarly, the analysis showed that CFB may be involved in the pathogenesis of NMOSD. Using the KEGG pathways analysis, it was determined that complement and coagulation cascades are associated with C2-CFB locus, previous studies showed AQP4 antibodies and complement-mediated damage are associated with NMOSD. Additionally, terminal complement inhibitor therapy of NMOSD has been proposed and proved effective among AQP4-IgG-positive NMOSD patients (Pittock et al., 2019). Similarly, the GO analysis demonstrated that regulation of humoral immune response (GO:0002920), protein processing (GO:0016485), regulation of complement activation (GO:0030449), regulation of protein activation cascade (GO:2000257) and complement activation (GO:0006956) are associated with C2-CFB locus. Based on molecular function, serine-type endopeptidase activity (GO:0004252), serine-type peptidase activity (GO:0008236) and serine hydrolase activity (GO:0017171) are associated with C2-CFB locus. Hence, our analysis shows that complements associated with C2-CFB locus play vital roles in the pathogenesis of NMOSD. However, in Estrada et al’s. (2018) study, C4 CNV was associated with NMO-IgG+ but not NMO- IgG-, which is slightly different.

HLA-DQA1 (MHC class II, DQ alpha 1) gene belongs to a group of MHC genes that encode the HLA-DQ heterodimer. Previous studies indicated that HLA-DQA1 is strongly associated with SLE (Demirci et al., 2016), systemic sclerosis (SSc) (Guo et al., 2016) and anticitrullinated protein antibodies (ACPA)-positive RA (Guo et al., 2019). Estrada et al’s. (2018) study demonstrated that HLA-DQA1 (rs28383224) was shared between AQP4-positive and negative NMOSD, suggesting that at least one common genetic determinant exists for both groups. However, in our study, there were no shared genes between the AQP4-positive and negative group. Given the complexity of the MHC region, larger studies will be needed to determine the role of HLA alleles to understand the effect of these haplotypes on the NMOSD subgroup.

In AQP4-positive NMOSD, SLE (hsa05322) was the most significant pathway (*p* = 6.62E-05) associated with HLA-DQA1, HIST1H2BL, HIST1H2AL, C2, and HIST1H3J locus. Other immune diseases, such as RA (hsa05323) were associated with HLA-DQA1 and ATP6V1G2 locus and it was also revealed that autoimmune diseases may coexist with NMOSD or share common pathological pathways. In previous studies, NMOSD with AQP4-IgG^+^ has been shown to be associated with a high frequency of autoantibodies and autoimmune diseases including SLE (Asgari et al., 2018), RA, Sjogren’s syndrome (SS) (Pittock et al., 2008), myasthenia gravis (MG) (McKeon et al., 2009), and antiphospholipid syndrome (APS) (Guerra et al., 2018). Regarding Estrada et al’s. (2018) study, NMO-IgG+ is genetically similar to SLE. Additionally, KEGG pathway analysis demonstrated *staphylococcus* (S.) *aureus* infection (hsa05150) to be associated with C2, CFB, and HLA-DQA1 locus was the second most significant signal (*p* = 8.17E-04) in AQP4-positive NMOSD. Previous studies showed that superantigens (SAgs) might be potent T cell activators, and it causes the deregulation of the immune response resulting in the proliferation of autoreactive T cells and the development and/or exacerbation of chronic autoimmune diseases (Foster, 2005). Among bacterial superantigens, the relevance of *S. aureus* superantigens with MS exacerbation has been depicted (Mulvey et al., 2011; Libbey et al., 2014). Kumar et al. demonstrated the beneficial effect of chronic *S. aureus* infection in a model of MS through the secretion of extracellular adherence protein, indicating a dual role of *S. aureus* infection in the pathogenesis of MS (Kumar et al., 2015). Our study revealed that the infection disease pathway might be involved in the pathogenesis of AQP4-positive NMOSD.

The MSH5 (MutS homolog 5) gene located in MHC class III region comprises 26 exons and spans 25 kb and is involved in DNA mismatch repair and meiotic recombination (Clark et al., 2013). Prior studies confirmed MSH5 as susceptibility loci for SLE, SS, and MS (Song et al., 2013; Demirci et al., 2016; Shen et al., 2019). Additionally, Fernando et al. determined the presence of risk and protective signals in and surrounding MSH5 (best risk SNP rs3130490; best protective SNP rs409558) in SLE (Fernando et al., 2012). According to the GO analysis, DNA repair complex (GO:1990391) and damaged DNA binding (GO:0003684) associated with MSH5 locus might be involved in the pathogenies of NMOSD. Previous studies showed defective DNA repair in SLE and even in quiescent SLE (Souliotis et al., 2016). Luo et al. revealed that novel autoantibodies were most related to cell death, cell cycle, and DNA repair pathways in SLE (Luo et al., 2019). Clark et al. highlighted important mechanisms of DNA methylation in RA and the wider context of immune dysregulation (Clark et al., 2020). Prior studies identified aberrant expression of apoptosis and DNA damage regulatory genes in MS, suggesting that DNA methylation may be effective in neuronal loss in RRMS (Gökdoǧan Edgünlü et al., 2020). Consistent with previous studies, MSH5 might contribute to the pathogenesis of NMOSD.

Euchromatic Histone Lysine Methyltransferase 2 (EHMT2) is a methyltransferase and is involved with the demethylation of histone H3 at lysine 9 (H3K9) (Fiszbein et al., 2016). *In vitr*o studies presented that EHMT2 promoted neuronal and immature oligodendrocyte differentiation was required for oligodendrocyte maturation. In previous studies, the methylation of histone H3K9, catalyzed by EHMT1 and EHMT2, was most depleted in patients with anti-neutrophil cytoplasmic autoantibody (ANCA)-associated vasculitis (AAV) (Yang et al., 2016). Ding et al. presented EHMT2 as a potent positive regulator of FOXP3 expression, playing essential roles in controlling immune responses in autoimmune diseases (Ding et al., 2019). A recent analysis observed higher C3 and lower EHMT2 plasma levels related to increasing brain atrophy in MS patients (Malekzadeh et al., 2019). The EHMT2 inhibitor triggered the inhibition of human diffuse large B-cell lymphoma cell proliferation leading to G1 phase arrest and induced apoptosis via endogenous and exogenous apoptotic pathways (Xu et al., 2021). The histone modification is a critical factor in regulating gene expression. Recent studies in autoimmune diseases suggest that increased expression of TLR2 in CD4^+^ T cells enhances immune reactivity and promotes IL-17 expression through upregulating H3K4 while downregulating H3K9 tri-methylation level in SLE (Liu et al., 2015). Additionally, Ding et al. discovered that increased B cell lymphoma 6 protein upregulates H3K27 trimethylation and downregulates H3K9/H3K14 acetylation of the MicroRNA-142 promoter in SLE CD4^+^ T cells (Ding et al., 2020). Araki et al. suggests that the histone lysine methylation (HKM) modifying enzymes results in changes of the gene expression of RA synovial fibroblasts (Araki et al., 2018). With respect to the GO analysis, histone H3K9 methylation (GO:0051567) and histone methyltransferase activity (H3K27 and H3K9) (GO:0046976 and GO:0046976), associated with EHMT2 locus, might be involved in the pathogenies of NMOSD.

The SLC44A4 (solute carrier family 44 member 4) gene located at 6p21.33 encodes human thiamine pyrophosphate transporter (TPPT), which is involved in the uptake of choline by cholinergic neurons. The SLC44A4 gene was reported to be associated with ulcerative colitis (UC) susceptibility in the Indian, Japanese, and Chinese populations (Gupta and Thelma, 2016; Gupta et al., 2016; Wu et al., 2017).

There were no shared potential susceptibility genes between the AQP4-positive and negative group. Given data limitations and the complexity of the MHC region, Further studies will be needed to understand the genetic and molecular mechanism underlying NMOSD.

## Conclusion

In AQP4-positive NMOSD cases, five shared risk genes were obtained at chromosome 6 using stringent *p*-Values in both methods (*p* < 0.05/16,532) comprising CFB, EHMT2, HLA-DQA1, MSH5, and SLC44A4. The 50 potential susceptibility gene sets were determined and 12 significant KEGG pathways were identified. In GO annotations of the 128 pathways identified, 67 biological process pathways, 32 cellular-component pathways, and 29 molecular-function pathways with a *p*-Value of <0.05 were obtained. There were no shared potential susceptibility genes between the AQP4-positive and negative group, also 4 significant KEGG pathways were identified. In the GO annotations of the 165 pathways identified, 99 biological process pathways, 37 cellular-component pathways, and 29 molecular-function pathways with a *p*-Value of <0.05 were obtained. The potential molecular mechanism underlying NMOSD may be related to proteins encoded by the novel genes in complements, antigen presentation, and immune regulation. The results may represent an improved understanding of the genetic and molecular mechanism underlying NMOSD.

## Data Availability Statement

The datasets presented in this study can be found in online repositories. The names of the repository/repositories and accession number(s) can be found in the article/Supplementary Material.

## Author Contributions

TL, HL, and C-SY conceived and designed the study for NMOSD. TL and HL analyzed the GWAS data and wrote the manuscript. LY and F-DS were responsible for subject guidance. C-SY was responsible for research supervision and manuscript revision. YL, S-AD, MY, Q-XZ, and BF provided technical support. All authors approved the final version for submission.

## Conflict of Interest

The authors declare that the research was conducted in the absence of any commercial or financial relationships that could be construed as a potential conflict of interest.

## Footnotes

1 https://www.ebi.ac.uk/gwas/downloads/summary-statistics

2 https://vegas2.qimrberghofer.edu.au/

3 http://pngu.mgh.harvard.edu/purcell/plink/

4 https://string-db.org/cgi/input.pl?sessionId=tigYoq2edJiZ&input_page_show_search=on

5 http://www.webgestalt.org/option.php

## Abbreviations

NMOSD: Neuromyelitis optica spectrum disorder
AQP4: aquaporin 4
GWAS: large-scale genome-wide association studies
VEGAS2: Versatile Gene-based Association Study-2 version 2
SNP: single nucleotide polymorphism
MHC: major histocompatibility complex
KEGG: Kyoto Encyclopedia of Genes and Genomes
GO: Gene Ontology
LD: linkage disequilibrium
MS: multiple sclerosis
SLE: systemic lupus erythematosus
RA: Rheumatoid arthritis
ATD: Autoimmune thyroid disease
SSc: systemic sclerosis
KD: Kawasaki disease
SS: Sjogren’s syndrome
MG: myasthenia gravis
APS: antiphospholipid syndrome
CSF: cerebrospinal fluid
MPs: microparticles
SPMS: secondary progressive MS
GPIb α: glycoprotein Ib α
EAE: Experimental Autoimmune Encephalomyelitis
CNS: central nervous system
RRMS: relapsing remitting multiple sclerosis.
